# Thermosensitive Polyurethane-Based Hydrogels as Potential Vehicles for Meloxicam Delivery

**DOI:** 10.3390/ph16111510

**Published:** 2023-10-24

**Authors:** Ioana-Alexandra Plugariu, Luiza Madalina Gradinaru, Mihaela Avadanei, Irina Rosca, Loredana Elena Nita, Claudia Maxim, Maria Bercea

**Affiliations:** 1 “Petru Poni” Institute of Macromolecular Chemistry, 41-A Grigore Ghica Voda Alley, 700487 Iasi, Romania; plugariu.ioana@icmpp.ro (I.-A.P.); mavadanei@icmpp.ro (M.A.); rosca.irina@icmpp.ro (I.R.); lnazare@icmpp.ro (L.E.N.); 2 Faculty of Chemical Engineering and Environmental Protection “Cristofor Simionescu”, “Gheorghe Asachi” Technical University of Iasi, 73A, D. Mangeron Blvd., 700050 Iasi, Romania; claudia.maxim@student.tuiasi.ro

**Keywords:** polyurethane, hydrogels, molecular docking, viscoelasticity, meloxicam delivery

## Abstract

Meloxicam (MX) is a nonsteroidal anti-inflammatory drug (NSAID) used mainly to reduce pain, inflammation, and fever. In the present study, thermosensitive polyurethane (PU)-based hydrogels with various excipients (PEG, PVP, HPC, and essential oil) were prepared and loaded with MX. Rheological investigations were carried out on the PU-based formulations in various shear regimes, and their viscoelastic characteristics were determined. The average size of the PU micelles was 35.8 nm at 37 °C and slightly increased at 37 nm in the presence of MX. The zeta potential values of the hydrogels were between −10 mV and −11.5 mV. At pH = 6 and temperature of 37 °C, the formulated PU-based hydrogels loaded with MX could deliver significant amounts of the active substance, between 60% and 80% over 24–48 h and more than 90% within 2 weeks. It was found that anomalous transport phenomena dominated MX’s release mechanism from the PU-based networks. The results are encouraging for further studies aiming to design alternative carriers to commercial dosage forms of nonsteroidal anti-inflammatory drugs.

## 1. Introduction

Nonsteroidal anti-inflammatory drugs (NSAIDs) are able to reduce pain and inflammation in the treatment of degenerative joint diseases such as arthritis or osteoarthritis (short-term treatment) and rheumatoid polyarthritis or ankylopoietic spondylitis (long-term treatment), and they are used in different pharmaceutical forms, including oral, injected, and topical dosage administration [1,2]. However, orally administered NSAIDs have shown many adverse side effects; they increase the risk of adverse reactions in the gastrointestinal tract [3], cardiovascular or renal failure, osteoporosis, hypophyse and hypothalamus suppression, and water and salt retention [4]. Their long-term use diminishes the life expectancy of patients with rheumatoid arthritis, a chronic inflammation of the synovial tissues affecting cartilage and joints [1].

Meloxicam (MX) is an analgesic and anti-inflammatory drug (NSAID) (4-hydroxy-2-methyl-N-(5-methyl-2-thiazolyl)-2H-1,2-benzothiazine-3-carboxamide-1,1-dioxide) with the ability to selectively inhibit cyclo-oxygenase-2 (COX-2) involved in the inflammatory response. Doses of up to 15 mg MX/day are usually recommended for the treatment of rheumatoid arthritis and osteoarthritis [5,6], with lower therapy costs compared with other NSAIDs (diclofenac or piroxicam) [7].

The main difficulty in the incorporation of MX in suitable delivery vehicles is its poor solubility in water. The prolonged transdermal delivery of MX still represents a challenge. Many efforts have focused on finding a suitable way to avoid the aforementioned gastrointestinal effects and promote local analgesia, providing steady levels in plasma [8,9]. Research in this field has been oriented toward the design of advanced drug delivery systems able to minimize the side effects during the administration of MX doses. This can be achieved by using either injectable or transdermal delivery systems [10,11,12]. Different organogels (glyceryl monostearate or glyceryl fatty acid ester) and hydrogels based on carbopol 940 or Pluronic F127 were investigated as transdermal delivery systems of MX using oleic acid, Mygliol 812, or Labrasol in a liquid phase [9]. Good release results and anti-inflammatory activity were obtained using organogels (Mygliol 812 and Labrasol). In situ gelling systems, such as Pluronics, can be considered effective vehicles for the delivery of poorly water-soluble drugs [12,13,14]. Below the lower critical solution temperature (LCST), they behave as liquid-like systems. Above the LCST, hydrophobic interactions increase, determining the formation of micelles with a hydrophilic core and a hydrophobic shell; further temperature and concentration increases make the entropy of mixing unfavorable, and micelle interactions can lead to fast sol–gel transitions [15,16]. Pluronics (F127 and F68, with a concentration of 20–30% in water, *w/w*) in the presence of different additives (poly(vinylmethylether-*co*-maleic anhydride), hydroxypropyl methylcellulose (HPMC), poly(ethylene glycol) 400, dimethyl sulfoxide, and sodium chloride) were tested, and it was found that Pluronic^®^ F127 in combination with HPMC could be considered as a vehicle for MX delivery [13]. However, this formulation is homogeneous at room temperature and tends to precipitate at body temperature. Other disadvantages of these smart temperature-responsive hydrogels (limiting their potential use in drug delivery applications) are their weak mechanical properties, poor biocompatibility, and delayed responses in physiological conditions [17].

Meloxicam was fully released within 20 days from hydrogels containing carboxymethyl chitosan, methylcellulose, and Pluronic. A slower release (in about 37 days) was obtained from hydrogels containing nanoparticles [11]. The cumulative release of MX was about 85% at pH 7.4 within 96 h from the alginate–chitosan–Pluronic composite nanoparticles [10]. Also, nanostructured lipid carriers were used for the enhancement of the transdermal absorption of MX [18].

Polyurethanes (PUs) are a versatile class of polymers that have been used in a wide range of applications [19,20,21,22]. Their versatility comes from their chemistry, which allows for the incorporation of various segments and specific functional groups into a polymeric backbone that enables adjustments of their properties. Furthermore, this versatility also opens the path to the design of novel PU-based hydrogels that are able to provide sustained and prolonged drug release or to be used as injectable materials in minimally invasive medicine [23,24,25,26]. The use of an amphiphilic polymer as a macrodiol in PU synthesis led to the preparation of stimuli-sensitive PU structures that could be further engineered to develop new formulations with enhanced properties. A poly(ethylene oxide)/poly(propylene oxide)/poly(ethylene oxide) (PEO–PPO–PEO) triblock copolymer (e.g., Pluronic P123) was selected as a macrodiol for the synthesis of PU due to its ability to undergo a lower critical solution temperature. As a result, these aqueous solutions exhibited a sol–gel transition with an increasing temperature (up to physiological values), making them attractive alternative drug delivery systems. Moreover, to further expand the promising biomedical properties of the resultant formulation, an aliphatic diisocyanate (L-lysine ethyl ester diisocyanate) was used.

The aim of the present study was to develop various formulations containing a thermosensitive PU structure and different excipients that could be used for therapeutic agent delivery. In this regard, a nonsteroidal anti-inflammatory drug (MX) was selected, and its release in correlation with gel characteristics was investigated.

## 2. Results and Discussion

A polyurethane (PU) sample was synthesized and then used as the main component for the development of thermosensitive hydrogels loaded with meloxicam (MX), an anti-inflammatory drug. Our main interest was in preparing PU-based samples in the sol state (low viscosity at a low temperature) for the facile incorporation of drug molecules. Under physiological conditions, the samples undergo gelation, leading to the formation of stable physical networks suitable for topical drug delivery. The pharmacokinetic benefits of these temperature-sensitive hydrogels for NSAID loading and delivery may be significant because a depot formulation can be generated and an anti-inflammatory drug (MX in our study) can be gradually eluted, preserving a constant local concentration of a drug in the surrounding tissues for a long period of time. In addition, they are very versatile, and their networks can be formed in situ, making them suitable for systemic delivery [26,27,28].

The PU-based formulations were prepared by using a mixture of 9.9 mL physiological serum (concentration of 0.15 M NaCl in aqueous solution) and 0.1 mL solution of NaOH (0.125 M NaOH in water), assuring the complete dissolution of MX. The gradual drug delivery at the targeted site depends on the excipients used. Klucel^®^ hydroxypropylcellulose (HPC) [29] was selected as the main excipient. HPC is a water-soluble cellulose ether that exhibits hydrophilic and hydrophobic interactions, having a lower critical solution temperature (LCST) around 41 °C [30]. It was previously reported that the gel consisting of 2.5% HPC, 0.3% MX, and 5% menthol in propylene glycol:ethanol:water = 1:1:1 can deliver therapeutically relevant doses of MX through the human skin [31]. In this study, two drug adsorption enhancers were used, namely poly(ethylene glycol) (PEG) and poly(vinylpyrrolidone) (PVP), known for their ability to improve the skin permeability [32,33,34,35]. The addition of these penetration enhancers influences the thermodynamic activity of MX in the delivery system [31]. PVP is a suitable additive that reduces the particle size to nanometers and prevents the aggregation of nanoparticles, increasing the MX solubility [33,36,37]. PEG is a biocompatible polymer able to enhance the dissolution rate of poor water-soluble drugs due to its hydrophilic and lipophilic properties [38,39].

Also, *Origanum Compactum* essential oil (EO) was incorporated as a skin penetration enhancer for transdermal MX delivery [40]. In addition, EO presents antimicrobial activity [41].

At low temperatures (below 20 °C), the PU-based samples (Table 1) presented liquid-like behavior. This is an important advantage for homogeneous loading of the poor soluble drug into the polymer solution. At physiological temperature, they became gels with solid-like behavior, containing well-dispersed MX into the polymeric matrix. These hydrogels were stable for a long period of time (they were observed for approx. 6 months), being of interest for topical formulations. A syneresis phenomenon occurred for Sample 3 stored for several weeks at room temperature. As a result, a contraction of the gel and an exclusion of a small amount of liquid phase took place. However, when the sample was vigorously mixed, the liquid was again incorporated into the polymer network. We suppose that the hydrophobic environment and dehydration of the PEO corona due to the interactions with PVP are responsive for this behavior [33].

The pH value of the normal skin surface is between 4.5 and 6.0 [42]. It was previously shown that the topical products should be slightly acidified, with an optimum pH value ranging from 4 to 6 [43,44]. Thus, for the present study, the value of pH = 6 was selected.

### 2.1. FTIR Spectra

**Figure 1:**
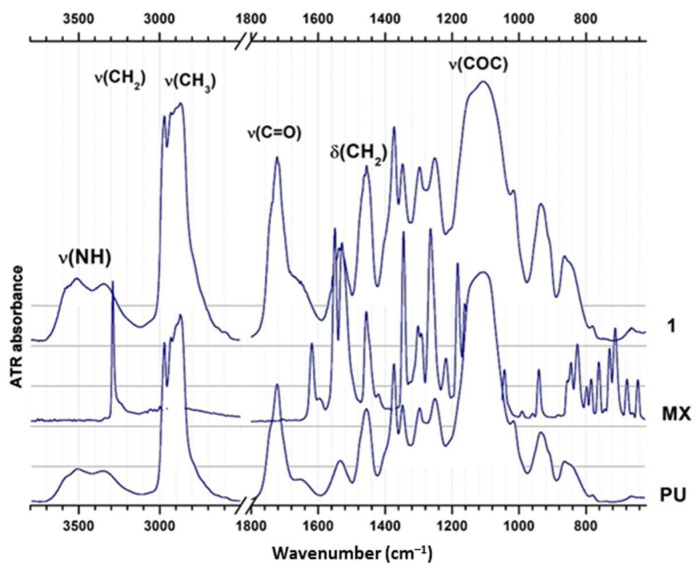
ATR-FTIR spectra of the starting compounds (PU and MX) and of the PU loaded with MX (Sample 1).

Figure 1 presents the FTIR spectra of PU, MX, and PU loaded with MX.

The first aspect to point out when making a comparative analysis between the infrared spectrum of the PU and Sample 1 is the high similarity between them, where most bands have the same profile and position. The signals of MX are overlapped by PU’s, but nevertheless, some spectral changes in PU may be observed. The ν(NH) region is structured and contains two absorption bands assigned to free –NH at 3585 cm^−1^ and 3514 cm^−1^. While only the first band is slightly reduced in intensity, it can be deduced that the most available urethane groups for interactions with MX were those located in the interphase. Based on the band intensity, this relocation would involve approx. 23% from the free urethane groups. The vibration of –NH groups that are intermolecularly H-bonded with C=O (3345 cm^−1^) is not changed.

The deformation and bending vibrations of methyl and methylene groups are also observed as being unchanged at 1453 cm^−1^, 1372 cm^−1^, or 1348 cm^−1^. The ester carbonyl stretching ν(C=O) at 1723 cm^−1^ has a lower intensity than in the initial PU, while the absorption band centered on 1110 cm^−1^ is the most affected. This fact indicates a possible redistribution of the intermolecular interactions between the hard segment of PU (represented mainly by ν(C=O)) and the Pluronic soft segment, which defines the maximum at 1110 cm^−1^. More precisely, the main peak at 1723 cm^−1^ belongs to free, non-associated C=O groups that could be located in the “disordered” interphase or in the soft regions. For Sample 1, the ν(C=O) band decreased with the simultaneous development of a sub-band around 1680 cm^−1^. This indicates that, for a fraction of carbonyls located in the interfacial regions, the bonding partner of the –N–H group has changed. Moving from a moderate to a strong –N–H ∙∙∙ H-bond implies that the ether oxygen atom from the Pluronic segment has been replaced by a carbonyl or sulfone oxygen from MX. The addition of MX into the PU matrix would lead to the breaking of some intramolecular C–H ∙∙∙ O and S–O ∙∙∙ H–C H-bonds in MX [45], and it would build intermolecular links with donor or acceptor groups from the PU matrix, such as C=O ∙∙∙ O–S and C=O ∙∙∙ H–N (MX). The other carbonyl bands observed around 1740 cm^−1^ (ester carbonyl from the lateral chain of the LDI segment and/or free urethane groups) and 1660 cm^−1^ (“ordered”, strongly H-bonded urethane C=O ∙∙∙ H–N–) have a similar intensity to the native PU.

**Figure 2:**
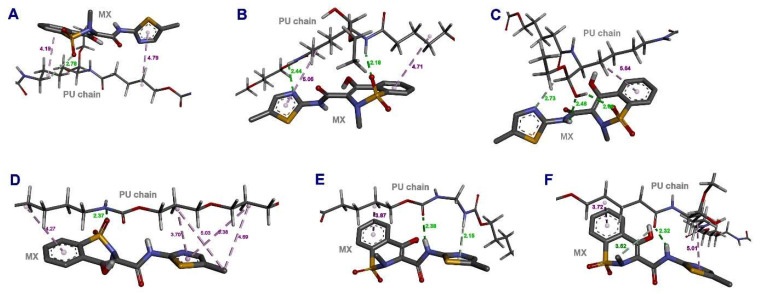
Docking model of the PU–MX matrix, showing the most favorable docking poses of MX: (**A**–**C**) involving the sulfone group of MX; (**D**–**F**) mainly hydrophobic interactions at the level of aromatic rings of MX. The two compounds are shown in the stick representation. Color code: magenta—hydrophobic interactions; green—hydrogen bonding. The distance between the donor and acceptor is given in Å.

The complex band centered at 1110 cm^−1^ is the envelope derived from the ester *ν*(C=O–O) of the hard segment and also from the composite vibration ν_s_(COC) + ν(CC) + ν_as_(COC) in the Pluronic soft segment. Although this band has roughly the same profile as in the initial PU, its lower intensity corresponds to the decrease of ν(C=O) band. This confirms that a part of the interactions between the urethane groups and the Pluronic ether groups have changed. The stable PU–MX matrices were identified by molecular docking, with the binding affinities between −2.9 and −3.1 kcal/mol. The most probable interactions between the PU matrix and MX can be summarized in Figure 2.

The addition of PEG into PU matrix (Sample 2) leads to a significant enhancement of the whole ν(NH) band due to the development of a new band around 3430 cm^−1^ at the expense of the free –NH groups from 3585 cm^−1^ and of strongly H-bonded groups from 3345 cm^−1^. This change accompanies the redshift of the ν(C=O) vibration to 1717 cm^−1^ and its decrease in magnitude, simultaneously with the appearance of new bands around 1665 cm^−1^, 1630 cm^−1^, and 1605 cm^−1^. It can be supposed that some intermolecular associations in the interfacial and disordered regions of PU have been broken upon PEG insertion between the chains. There is a fraction of N–H ∙∙∙ O< (PEG) links of moderate strength (3430 cm^−1^) and several kinds of urethane groups with either H-bonded C=O (1630 cm^−1^) or with free C=O and N–H ∙∙∙ O=C from an adjacent chain (1665 cm^−1^). The urethane units having the free C=O and N–H ∙∙∙ O< (PEG) would absorb at 1717 cm^−1^.

The infrared spectra of Samples 4 and 5 share different kinds of ν(NH) (3420 cm^−1^) and ν_s_(COC) (Pluronic) + *ν*(C=O–O) (urethane) (1095 cm^−1^) bands, due to the contribution of the specific signals of HPC. The free ν(NH) component is missing in both hydrogels. The main ν(C=O) peak is downshifted to 1720 cm^−1^ and a new absorption between 1650 and 1580 cm^−1^ appears, suggesting a reorganization of the strongly H-bonded carbonyls. Involvement of a fraction of urethane –NH groups in the association with –OH groups of HPC also affected the vibration of the C=O bond, lowering it by several cm^−1^. The lower position of the ν_s_(COC) + *ν*(C=O–O), shifted to 1095 cm^−1^, is a combination of both Pluronic and HPC maxima. This fact suggests the high compatibility between them, involving a reordering of the Pluronic chains, and the high degree of mixing at the level of PU soft segments. According to the literature, the PVP addition determines a weak secondary bonding between MX and PVP (according to FTIR spectra presented in Figures 6 and 7 from Ref. [34]), whereas MX exhibits a high and fast dissolution rate in PVP-based formulations [33,37].

### 2.2. Rheological Behavior

#### 2.2.1. Temperature-Induced Gelation Monitored by Rheological Measurements

The rheological measurements were used to delimit the temperature ranges where the samples are in a sol state or gel state for PU-based samples and to characterize the gel behavior at a physiological temperature. Thus, the elastic (*G*′) and viscous (*G*″) moduli, as well as the loss tangent (*tan δ* = *G*″/*G*′), were monitored in various oscillatory tests as a function of temperature, strain amplitude (*γ*), oscillation frequency (*ω*), or time.

**Figure 3:**
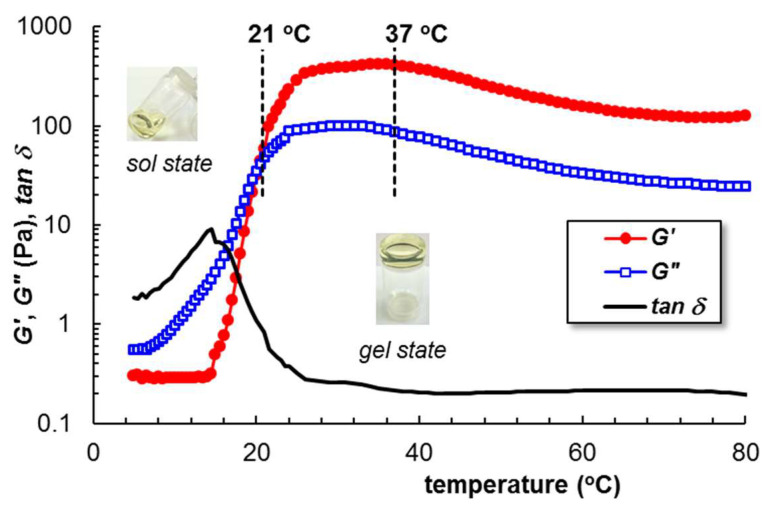
The viscoelastic parameters *G*′, *G*″, and *tan δ* as a function of temperature for PU sample loaded with MX and submitted to a heating rate of 0.5 °C/min (*γ* = 1%, *ω* = 10 rad/s).

Sample 1 is in a sol state at low temperature, with a predominantly viscous behavior: *G*″ > *G*′ and *tan δ* > 1 (Figure 3). A sol–gel transition occurs around 21 °C, and above this temperature the solid-like behavior prevails. The temperature increase determines the intensification of hydrophobic interactions and the formation of micelles with the hydrophobic core and hydrophilic shell. The gelation of PU is attributed to the formation of a polymicellar network structure [46], when a sharp variation in the viscoelastic parameters takes place. In the gel state, *G*′ > *G*″ and *tan δ* < 1. The network strength is not significantly influenced by MX addition or the heating rate, but it is sensibly influenced by the excipients used in the gel formulation (Table 1).

**Figure 4:**
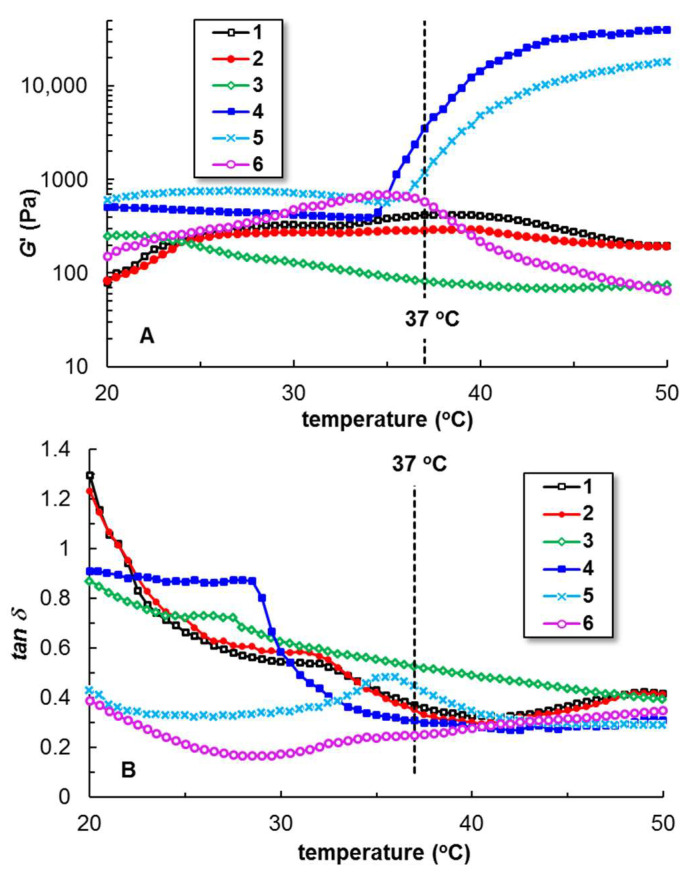
The temperature dependence of (**A**) elastic modulus (G’) and (**B**) loss tangent (*tan δ*) for the hydrogels loaded with MX, heating rate of 1 °C/min, *γ* = 1%, *ω* = 1 rad/s.

Figure 4 shows the rheological behavior of Samples 1–6 from 20 °C (close to the storage temperature) to 50 °C (above the physiological temperature). PEO and PVP weaken the network, *G*′ decreases, and *tan δ* increases for Samples 2 and 3. The behavior of Samples 4 and 5 is strongly influenced by HPC addition. The highly entangled HPC chains and PU micelles [46,47] form an interpenetrating network with preponderant hydrophilic interactions at low temperatures, ensuring the homogenization of the polymer mixtures in solution. By raising the temperature, the hydrophobic interactions are developed by both polymers and determine an increase in the viscoelastic moduli. However, above 41 °C, the loss tangent remains almost constant, regardless of the HPC content in the PU/HPC mixture. Sample 6 presents a compact and stable structure at low temperatures, with the oil molecules well dispersed into the amphiphilic micellar network of PU. The network strength decreases above 36 °C, probably due to the decrease in the intermolecular interactions between PU chains and oil molecules.

#### 2.2.2. Linear and Non-Linear Viscoelasticity

**Figure 5:**
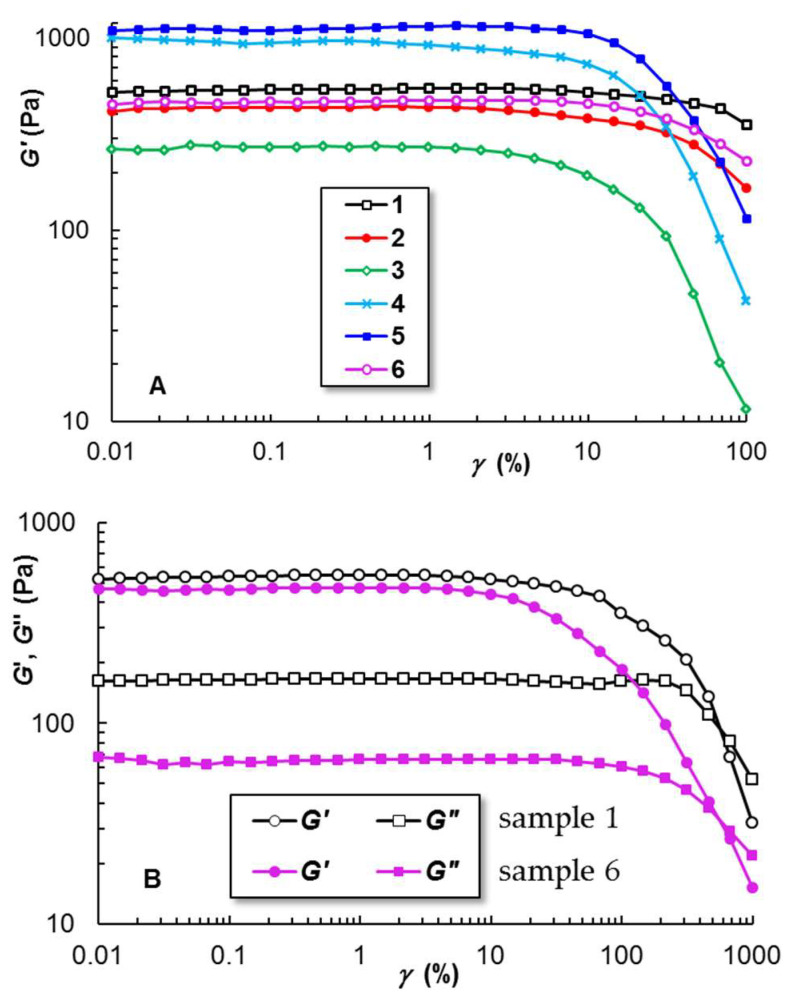
The dependence on the strain amplitude of (**A**) *G*′ for Samples 1–6; (**B**) *G*′ and *G*″ for Samples 1 and 6 (*ω* = 10 rad/s, 37 °C).

Amplitude sweep tests were carried out for all hydrogels loaded with MX in order to determine the limiting strain value ($\gamma_{L}$) (Table 1), at the end of the linear viscoelastic region (VLR), when the viscoelastic moduli start to decrease. For the PU-based hydrogels, $\gamma_{L}$ was estimated from *G*′–*γ* dependences (Figure 5A) because *G*″ decreases at higher *γ* values (Figure 5B). This can be due to the presence of salts that can influence the hydrophilic/hydrophobic balance. $\gamma_{L}$ is correlated with the stability of the hydrogel submitted to the action of the external forces. For $\gamma \;<\;\gamma_{L}$, *G*′ > *G*″ and both viscoelastic moduli are independent of strain; for $\gamma \;>\;\gamma_{L}$, the physical network is broken and the sample undergoes viscous flow (permanent and indefinite deformation). Higher $\gamma_{L}$ values suggest a higher network resistance to deformation.

#### 2.2.3. Self-Healing Behavior

**Figure 6:**
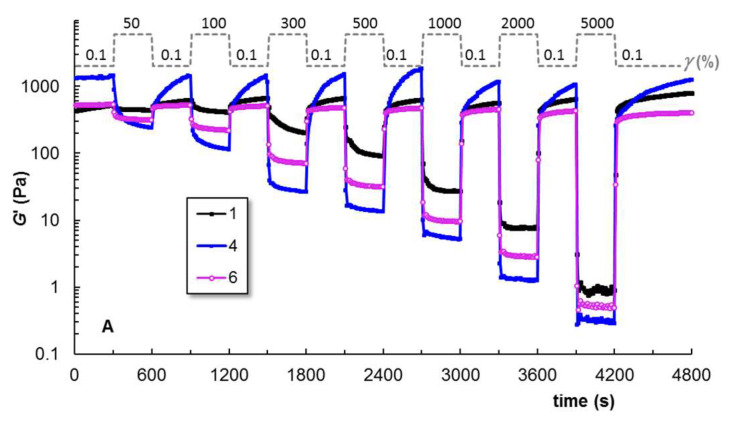

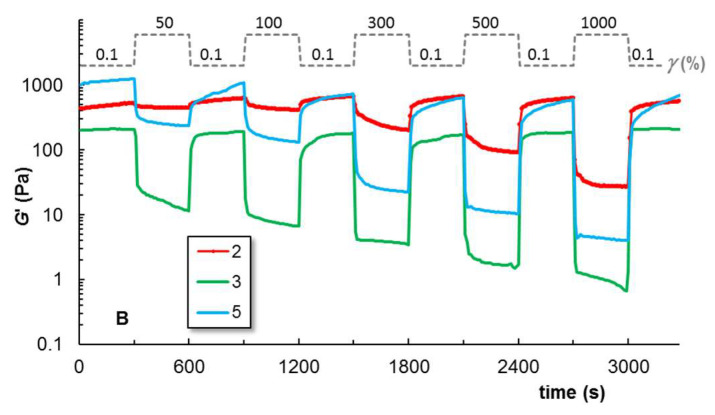
Illustration of self-healing behavior of hydrogels through variation of *G*′ during the experiments carried out at low (linear viscoelastic domain) and high (non-linear domain of viscoelasticity) successive step strains applied every 300 s: (**A**) Samples 1, 4, and 6; (**B**) Samples 2, 3, and 5.

The analysis of the structure recovery after applying large amplitude deformations allows us to discuss the ability of the hydrogel samples to exhibit self-healing behavior. Thus, successive cycles of low strain values (in the linear range of viscoelasticity, according to Figure 5) and high strains ($\gamma \;>\;\gamma_{L}$) were applied to the hydrogels and the viscoelastic parameters were monitored as a function of time (Figure 6).

For Samples 1, 2, and 6, the structure integrity is recovered very quickly (within seconds). In the presence of long HPC chains (Samples 4 and 5), the return to the previous G’ value (before applying high deformation) is slower and time dependent. The structure recovery is very fast for PU micelles (Sample 1), also in the presence of shorter PEO chains (Sample 2) or EO (Sample 6). The addition of PVP in the PU/PEO system (Sample 3) slightly influences the recovery time, but it determines a decrease in *G*′ and other viscoelastic parameters (Table 1). This can be explained by the interactions between C=O groups of PVP chains and C–O–C groups of PEO blocks from micelles corona, influencing the colloidal stability [48,49]. In our case, the increase of PVP content diminishes or even cancels the gelation of PU. PVP chains have a stronger enthalpy of adsorption to micellar corona as compared with PEG [48]; thus, the destabilization of the PU network structure is higher, as can be seen from rheological data. However, both excipients, PEG and PVP are recommended as adsorption enhancers for MX [33,37]. As a result, the composition of Sample 3 was optimized by incorporating a PEG/PVP mixture in the PU sol state. The intermolecular interactions between free PEG and PVP chains in an aqueous saline environment somehow moderate the effect of PVP on PU gelation at an increasing temperature. For Samples 1, 4, and 6, the structure is well recovered after applying high strains (2000% or 5000%, Figure 6A), but for Samples 2, 3, and 5 the network structure fails in the conditions of very high deformations (*γ* > 1000%).

#### 2.2.4. Shear Flow Behavior at 37 °C

**Figure 7:**
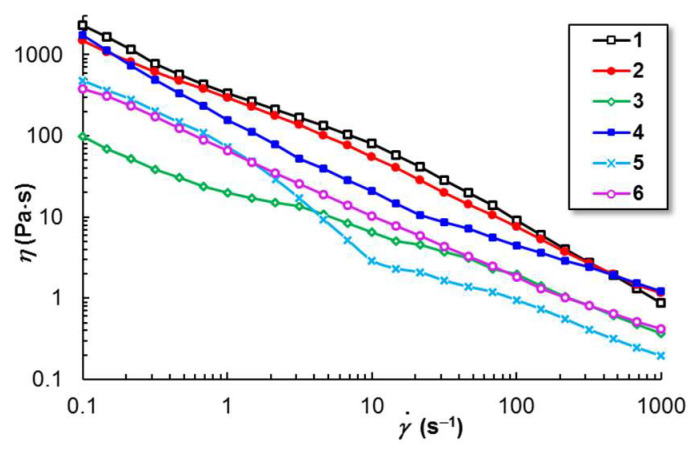
Shear viscosity as a function of shear rate for the hydrogel samples loaded with MX (at temperature of 37 °C).

Figure 7 shows the dependence of apparent viscosity (*η*) as a function of the shear rate ($\dot{\gamma }$) for the investigated gel samples. The flow is non-Newtonian and the viscosity scales as *η* ~ ${\dot{\gamma }}^{-n}$. The flow index *n* is between 0.78 and 0.82 for Samples 1, 2, and 6, and it takes the value of 0.6 for Sample 3. For $\dot{\gamma }$ > 10 s^−1^, a change in the slope (decrease in *n* value) was depicted for Samples 4 and 5. For a high HPC content, a pronounced viscosity decrease can be observed. This can be due to the high shear forces that overcome the strong hydrophobic interactions occurring in the presence of HPC chains. The drug release rate from the polymeric matrix is usually inversely proportional to its viscosity [28].

Generally, the yield stress fluids are able to deform like solids (finite deformation) and to flow only by applying a stress above a critical value [50]. From the equilibrium flow curves (stress-controlled experiments), the yield stress (*τ*_o_) was determined and its values for Samples 1–6 are given in Table 1.

#### 2.2.5. Creep and Recovery Behavior

**Figure 8:**
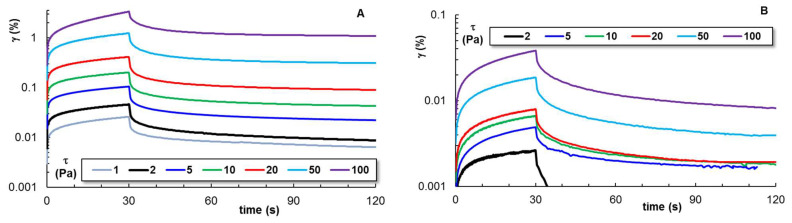

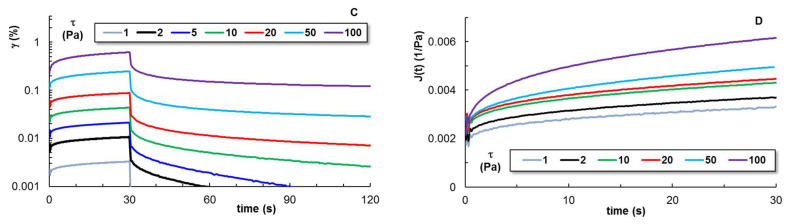
The strain evolution during the creep at different shear stress (*τ*) values applied for 30 s and the strain recovery (*τ* = 0) for (**A**) Sample 1; (**B**) Sample 4; (**C**) Sample 6; (**D**) creep compliance for Sample 6.

The analysis of the creep and recovery behavior also allows the evaluation of the viscoelasticity of the gels submitted to the action of a constant shear stress (*τ*) for 30 s (creep curves) through the strain *γ* (*t*) (Figure 8A–C) or compliance *J*(*t*) (Figure 8D) variation in time. When the shear stress is removed, the recovered strain is monitored until the equilibrium state is reached (recovery curves). The creep compliance is correlated with the strain as:

(1) $$J(t)=\frac{\gamma \;(t)}{\tau }$$

Below 100 Pa, the hydrogels undergo viscoelastic behavior (as shown in Figure 8 for Samples 1, 4, and 6). During the creep test (*τ* = 0), the transient response consists of fast (*γ*_el_) and delayed (*γ*_d_) elastic strains followed by a viscous flow (*γ*_v_). The recovery takes place in the same order: *γ*_el_–*γ*_d_–*γ*_v_, and the recovery process (the required time and the value of the irreversible component, γ_v_, at equilibrium) depends on the applied *τ* value. For *τ* < *τ*_o_, the creep compliance, *J*(*t*), decreases with increasing shear stress for PU hydrogels in the presence of other polymeric excipients (Samples 1–5). For *τ* > *τ*_o_, *J*(*t*) changes its trend and starts to increase due to the preponderance of the viscous flow. Similar behavior was reported for PU/peptide systems [46]. However, an anti-creep behavior was observed for Sample 6 (Figure 8D) due to the EO presence.

### 2.3. Zeta Potential and Average Diameter of PU Micelles

**Figure 9:**
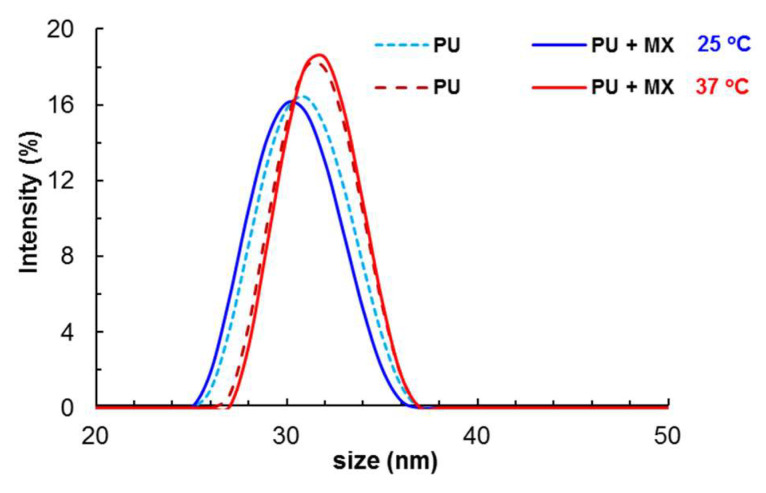
The size distribution for the PU micelles in the presence (continuous line) and absence (dashed line) of MX at 25 °C (blue line) and 37 °C (red line).

PU, PEG, and HPC used for hydrogel formulation are neutral polymers. Due to the ions resulting from the salts dissolved in an aqueous solution and used as a solvent for MX, the values of the zeta potential of the hydrogels are negative and range between −10 ± 0.3 mV and −11.5 ± 0.345 mV. The presence of MX determines a slow increase in the number of negative charges in the environment. In the solvent used in hydrogel formulation, the mean hydrodynamic diameter (*D*_H_) of PU micelles increases from 32.8 nm to 35.8 nm when the temperature increases from 25 °C to 37 °C. The interaction with MX contributes to a slight rise in the *D*_H_ value of PU micelles (Figure 9). A value of 37 nm was registered at 37 °C for PU in the presence of MX. The values of the polydispersity index (PDI) are below 0.4, indicating a low polydispersity and a monomodal distribution peak (Table 2).

It was shown that the drug loading capacity and therapeutic properties are improved when the network used as a carrier is composed of micelles with an average size lower than 200 nm [51]. Due to the nanomicellar structure of the PU matrix with a hydrophobic core and hydrophilic shell, these hydrogels are able to encapsulate both hydrophilic and hydrophobic compounds for the local delivery of different active principles in a single matrix. The incorporation of poor water-soluble MX into the PU nanomicellar hydrogels enhanced the solubility of the drug, bioavailability, and formulation stability.

### 2.4. Meloxicam Delivery from PU-Based Hydrogels

**Figure 10:**
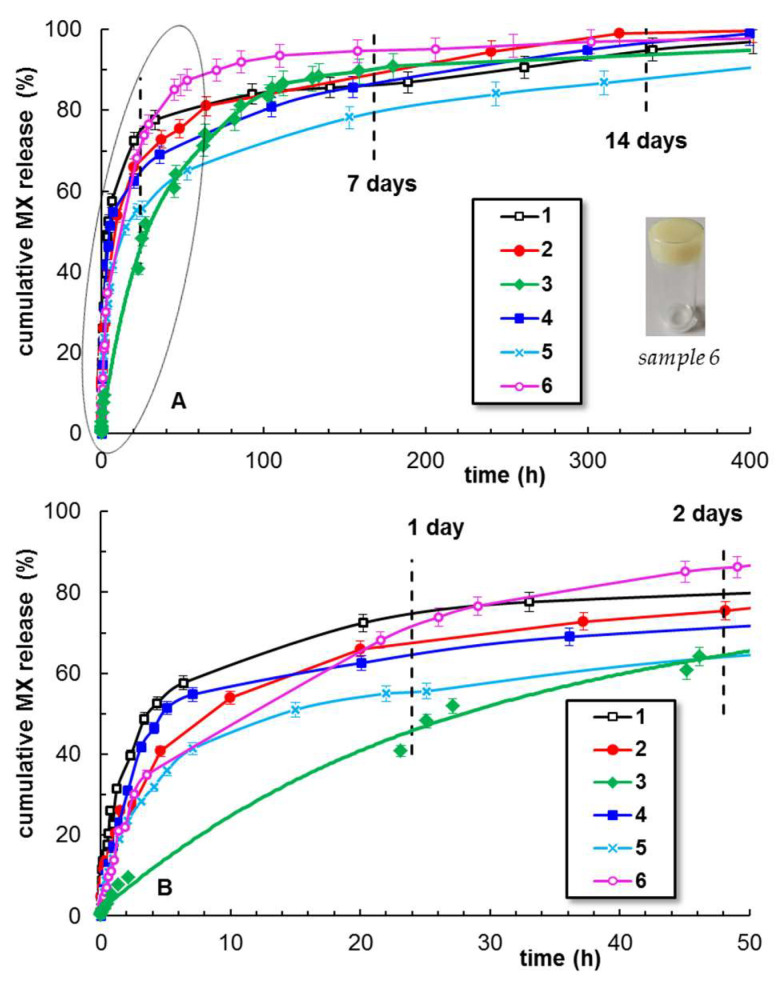
Kinetics of meloxicam delivery from PU-based hydrogels (37 °C, pH = 6): (**A**) cumulative release of MX during 400 h; (**B**) details of the release curves during the first 50 h (gray ellipse from Figure (**A**)). The lines are a guide to the eye.

Figure 10 presents the MX release profiles for the six hydrogel samples. The cumulative drug release during the first 24 h reaches levels between 40% and 70%, depending on the sample composition. The maximum efficiency is registered for Samples 1 (PU hydrogel) and 6 (PU hydrogel containing essential oil). These two formulations are suitable as gels for topical applications. The polymeric excipients addition improves the delivery profile for a longer period of time (Samples 2–4); thus, these gels could be considered appropriate materials for wound-dressing applications. Sample 5, with a high HPC content, releases less than 50% MX during 24 h. Then, a slow MX release is registered for two weeks until the delivered MX reaches about 80%.

Similar delivery profiles were reported for alginate–chitosan–Pluronic composite nanoparticles, with 85% of the cumulative MX released within 96 h at pH = 7.4. In such systems, the mean dissolution time was about 9 h [10]. The advantage of our formulation is the rapid dissolution of MX in the saline solutions and then the PU is easily dissolved at a low temperature (5 °C) in a sol state. In this way, the homogeneous distribution of the drug in the network is ensured during gelation. At room temperature, the gel is formed and the MX molecules remain immobilized in the gel, without any separation in time.

The MX delivery from the hydrogel samples can be discussed by using different mathematical models. Among them, first-order [52] and Higuchi [53] models allow the discussion of the delivery data through the first-order and Higuchi release rate constants, *k*_11_ and *k_H_*, respectively:

First-order:    *ln*(1 − *M_t_/M_∞_*) = −*k*_11_
*t* (2)

Higuchi:    *M_t_/M_∞_* = *k_H_ t*^1/2^ (3)

where *M_t_*/*M*_∞_ represents the fraction of the drug released at the time *t*.

The most versatile approaches were developed by Peppas and coworkers [54,55] and they are suitable for drug delivery from various hydrogels:

Korsmeyer–Peppas:    *M_t_*/*M*_∞_ = *k t^n^* (4)

Peppas–Sahlin:    *M_t_*/*M*_∞_ = *k*_1_ *t^m^* + *k*_2_ *t*^2*m*^ (5)

*k*, *k*_1_, *k*_2_ are the release rate constants, which depend on the matrix and drug characteristics. Equation (4), applied for the first drug-release periods until 60% of the maximum level is reached, gives the release exponent *n*. The *n* value allows the discussion of the drug-release mechanism from the network, i.e., the Fickian or non-Fickian contributions to the diffusion during the drug delivery process [55]. For the PU-based hydrogels in the presence or absence of excipients, 0.45 < *n* < 0.89 (Table 3), suggesting the occurrence of anomalous transport phenomena, controlled by the drug diffusion through the network [54,56,57].

Topical gels loaded with MX exhibit reduced systemic side effects and a better performance against pain and inflammation as compared with other NSAID gels (diclofenac or piroxicam) [58]. The homogeneous incorporation and delivery of poor water-soluble MX into PU-based hydrogels make them promising topical gels. The thermosensitive PU matrix (Sample 1) appears as a suitable carrier for short-term meloxicam delivery. The addition of a small amount of polymeric excipients (Samples 2–5) improves the long-term drug delivery. Furthermore, the incorporation of the essential oils (Sample 6) offers multiple other benefits in controlling the drug release, improving the biological activity and transdermal drug delivery [40,41,59], and reducing the ulcerative side effect associated with MX [60]. The selected essential oil was extracted from *Origanum Compactum* and presents antimicrobial and antifungal properties.

In conclusion, the developed PU-based systems can fulfill some of the basic requirements for a matrix that can easily encapsulate and efficiently deliver the anti-inflammatory drug. Also, the selected excipients can act as delivery enhancers or therapeutic agents, ensuring the optimal MX concentration over time or, in addition, can provide antimicrobial activity to the formulation. Further studies will be focused on improving the biological properties of the PU-based hydrogels, as alternative carriers to commercial dosage forms of nonsteroidal anti-inflammatory drugs.

## 3. Materials and Methods

### 3.1. Materials

#### 3.1.1. Chemicals

Poly(ethylene oxide)_20_–*b*–poly(propylene oxide)_70_–*b*–poly(ethylene oxide)_20_ (PEO–*b*–PPO–*b*–PEO), M_n_ = 5.8 × 10^3^ g/mol, poly(vinylpyrrolidone) (PVP), M = 4 × 10^4^ g/mol, and 1,4-butane-diol (BD) were purchased from Sigma Aldrich (Steinheim, Germany). L-lysine ethyl ester diisocyanate, 97%, (LDI) was obtained from Alfa Aesar (Kandel, Germany) and was freshly distilled before synthesis. Poly(ethylene glycol) (PEG), M = 4 × 10^2^ g/mol, sodium hydroxide (NaOH), and sodium chloride (NaCl) were purchased from Fluka Chemie GmbH (Switzerland). A sample of Klucel™ hydroxypropylcellulose (HPC) with M = 9.5 × 10^4^ g/mol was kindly offered by Ashland Aqualon [29].

The oregano essential oil used for preparing sample 6, extracted from *Origanum Compactum* (family *Lamiaceae*, species native from Morocco), was provided by Euflora Company (Iasi, Romania) and contains mainly carvacrol (53%), tymol (28%), paracymene (26%), and terpinene gamma (20%).

The meloxicam sample (99%, M = 351.4 g/mol) was purchased from Thermo Fisher Scientific (Leicestershire, UK).

**Scheme 1:**
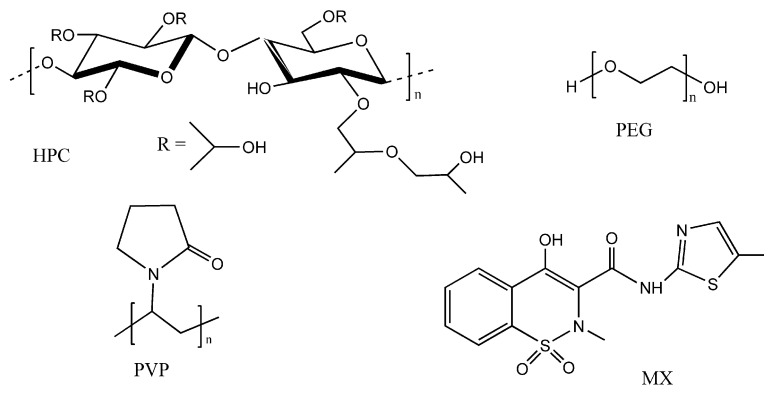
Structure of the commercial polymers and the investigated drug.

The structures of the commercial chemicals and drug used in the present study are given in Scheme 1.

#### 3.1.2. Synthesis of Thermoresponsive Polyurethane

**Scheme 2:**
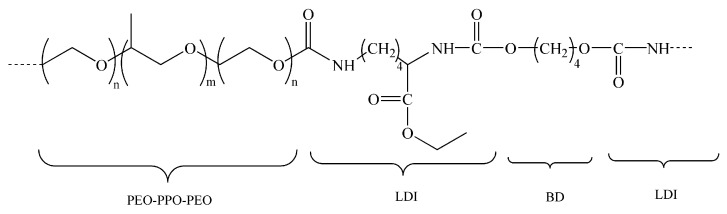
Structure of the synthetized thermoresponsive polyurethane.

The polyurethane (PU) sample was synthesized according to a procedure recently reported [46], via a standard two-step prepolymer method. In brief, the copolymer (PEO–*b*–PPO–*b*–PEO) sample was firstly dehydrated and then reacted with an excess of LDI for 4 h at 80 °C, without any catalyst. Then, the BD chain extender was added to the prepolymer and allowed to react for another period of 6 h. The reaction was carried out using an NCO:OH molar ratio of 1:1. The resulting PU structure is presented in Scheme 2.

#### 3.1.3. Preparation of Thermoresponsive PU-Based Hydrogels

Firstly, the appropriate amount of MX was dissolved in the physiological serum of pharmaceutical use (0.15 M NaCl aqueous solution) at room temperature. The pH was fine-adjusted by using an aqueous solution of 0.125 M NaOH until the complete dissolution of the drug (0.1 mL NaOH solution was required for 9.9 mL physiological serum). Then, the PU was added and the mixture was stored in the refrigerator and mixed from time to time by using a roller mixer at a low rotation speed. The excipients were added to the sample in the sol state and the final formulations were homogenized by further mixing and then stored at room temperature. The syneresis appearance (as observed for PVP containing sample stored for a long time) can be avoided by using a small amount of organic salts [61].

### 3.2. Methods

#### 3.2.1. Attenuated Total Reflectance-Fourier Transform Infrared Spectroscopy

ATR-FTIR spectra were recorded using a Bruker Vertex 70 type spectrometer (Bruker, Germany), equipped with a diamond ATR device (Golden Gate, Bruker, Germany) and software for spectral processing. Spectra were obtained in absorbance mode in the range of 4000−500 cm^−1^, averaging over 64 scans at a resolution of 2 cm^−1^.

#### 3.2.2. In Silico Investigations of Polymer-Drug Binding

The most probable binding sites of MX with the PU matrix have been explored through a ligand flexible docking study. The 2D model of the PU chain has been drawn in Marvin JS by Chemaxon [62]. Associated SMILES were used to build the 3D model and to optimize it by using USCF Chimera [63]. The input files for docking were prepared by using USCF Chimera and AutoDock Tools 1.5.7 packages (Molecular Graphics Lab, La Jolla, CA, USA). The blind docking experiment was performed with AutoDock Vina 1.2.0 [64]. The final pose of every docked MX has been selected from the top-binding conformations with the highest binding score. The PU–MX interactions were visualized and the structural figures were drawn in BIOVIA Discovery Studio [65].

#### 3.2.3. Rheology

Rheological measurements were carried out by using an MCR 302 Anton-Paar rheometer (Gratz, Austria), equipped with plane–plane geometry of 50 mm (the selected gap was of 500 µm) and a Peltier device for temperature control.

The temperature-induced gelation was monitored through the viscoelastic parameters, firstly for Sample 1 at a heating rate of 0.5 °C/min from 5 °C to 80 °C. Then, all samples were tested with a heating rate of 1 °C/min for the temperature range of interest (from 20 °C to 50 °C). The elastic (*G*′) and viscous (*G*″) moduli were registered as the amount of stored and dissipated energy during one cycle of deformation, respectively. The loss tangent (*tan δ* = *G*″/*G*′) expresses the degree of viscoelasticity: *tan δ* < 1–solid-like behavior and *tan δ* > 1–liquid-like behavior.

The gels thermostatted at 37 °C were investigated in various shear conditions. Amplitude sweep tests were carried out to determine the upper limit of strain, $\gamma_{L}$, for the linear viscoelastic regime (37 °C, ω = 10 rad/s). The thixotropic tests were carried out in oscillatory regime for *ω* = 10 rad/s and step strains successively changed every 300 s from low (0.1%, value of strain in the linear viscoelastic domain) to high values (50%, 100%, 300%, 500%, 1000%, 2000%, and 5000%, that belong to nonlinear range of viscoelasticity) and again at the low step of strain (0.1%). The shear viscosity was determined as a function of shear rate ($\dot{\gamma }$) from 0.1 s^−1^ to 1000 s^−1^. Yield stress (*τ*_o_) was determined from the stress-controlled experiments carried out in stationary shear flow conditions. The creep and recovery curves were monitored at different shear stress (*τ*) values applied for 30 s during the creep test and then, for *τ* = 0, the strain recovery was monitored over time.

#### 3.2.4. Zeta Potential and Average Diameter

Zeta potential (*ζ*) and hydrodynamic diameter (*D*_H_) values were determined by using Zetasizer Nano ZS instrument (Malvern Instruments, UK, equipped with 633 nm laser He/Ne and Peltier device), according to the following relationships:

(6) $$\zeta =\frac{\eta \;\mu }{\varepsilon }\;\;\;\;\;(\mathrm{Smoluchowski equation})$$

and

(7) $$D_{H}=\frac{k\;T}{6\;\pi \;\eta \;D}\;\;\;\;\;(\mathrm{Mie method})$$

where: *η* is the viscosity; *μ*—electrophoretic mobility; *ε*—dielectric constant; *k*—Boltzmann constant; *T*—absolute temperature; *D*—diffusion coefficient.

Each measurement was performed 3 times and the average values were considered.

#### 3.2.5. Meloxicam Delivery

The drug-loaded hydrogels were prepared by adding 1% wt. MX to the polymer solutions prepared as shown above (Section 3.1.3). In vitro drug release behavior in the physiological serum environment was evaluated by the dialysis method. About 0.5 g sample was introduced in a dialysis bag (the molecular weight cut-off being 10 kDa), immersed in a closed bottle containing 15 mL of salted solution (pH = 6), and placed in a thermostatic chamber at a constant temperature of 37 °C. At different time intervals, aliquots of 1 mL were withdrawn from the release medium and replaced by 1 mL pre-heated solution, in order to maintain a constant volume. The quantity of drug in the release medium was determined by UV spectrophotometry, using a standard calibration curve (a linear dependence of absorbance versus drug concentration was determined). The absorbance was recorded at 362 nm using a Libra UV–Vis spectrophotometer (Biochrom Libra S35PC, Cambridge, UK). The drug-release studies were performed in triplicate. The experimental data were analyzed using OriginPro 8.5 software and the values obtained for the minimum values of the residual sum of the squares (*RSS*) were considered [66]. However, the number of parameters (*p*) involved in a given model influences *RSS* values. The Akaike Information Criterion (AIC) [67] was used for the statistical analysis of the drug-release mechanism, being independent of the number of parameters introduced by each model:

AIC = *N* ln(*SSR*) + 2 *p* (8)

where *N* represents the number of experimental data.

According to this criterion, the model with the smallest AIC value is taken into consideration as the best fitting model to describe the drug-release mechanism.

## 4. Conclusions

In this study, PU-based hydrogel carriers were successfully developed, capable of incorporating specific functional groups and imparting new functionalities that can be exploited in the design of smart materials, used to ensure a sustained and prolonged drug release. Different formulations of the thermal-sensitive PU-based hydrogels loaded with MX were prepared as a minimally invasive alternative for drug administration. The influence of several excipients, such as HPC, PEO, PVP, and oregano essential oil, was analyzed. FTIR spectra revealed the established interactions and rheological data indicated some particular behaviors depending on the composition of the system. The delivery of the active substance in the physiological serum environment was investigated at 37 °C and the process was dominated by anomalous transport phenomena. The main conclusion is that the PU-based hydrogels containing only PU or PU in the presence of a small amount of PEG or essential oil (with antimicrobial activity) are suitable for reducing local inflammation and pain during a period of 24 h to 48 h. On the other hand, the addition of a PEO/PVP mixture or low HPC content allows the design of suitable MX vehicles for wound-dressing applications with extended anti-inflammatory drug release. The use of natural therapeutic agents (essential oils with antimicrobial activity) as excipients presents a high potential for the development of new PU-based carriers for MX delivery.

Further studies are required to analyze the ability to deliver therapeutically relevant amounts of MX and the evaluation of the anti-inflammatory effect induced by oil addition.

## Figures and Tables

**Table 1:** The composition of the investigated hydrogels and their rheological characteristics at 37 °C.

Sample Code	PU (% wt.)	Excipient Type	Excipient Content (% wt.)	Solvent (% wt.)	MX (%)	*τ*_o_ ^(a)^ (Pa)	$\;\gamma_{L}$ (%)	*G*′ ^(b)^ (Pa)
1	25	-	-	75	1	79.2	31.6	525.5
2	25	PEG	3	72	1	72.8	14.7	456.1
3	25	PEG PVP	3 3	69	1	9.4	3.2	208.6
4	18.75	HPC	6.25	75	1	87.3	10.9	1330
5	12.5	HPC	12.5	75	1	85.6	6.8	1203
6	25	EO	6	69	1	46.1	21.5	474.3

^(a)^ continuous flow (imposed shear stress experiment); ^(b)^ *ω* = 10 rad/s; *γ* = 0.1%; PU = polyurethane; MX = meloxicam; PEG = poly(ethylene glycol); PVP = poly(vinylpyrrolidone); HPC = hydroxypropylcellulose; EO = *Origanum Compactum*.

**Table 2:** The particle size (expressed as the mean hydrodynamic diameter, *D*_H_) and polydispersity index (PDI) for PU micelles and PU loaded with MX at 25 °C and 37 °C.

Sample	Temperature (°C)	*D*_H_ ± SD * (nm)	PDI
PU	25	32.8 ± 0.98	0.120
PU loaded with MX	25	31.6 ± 0.94	0.132
PU	37	35.8 ± 1.06	0.245
PU loaded with MX	37	37.0 ± 1.10	0.246

* SD is the standard deviation.

**Table 3:** The parameters that describe the kinetics of MX release from hybrid hydrogels according to Equations (2)–(5) (37 °C, pH = 6). The values of standard deviations were below ±3%.

Sample Code	First Order Equation (2)		Higuchi Equation (3)		Korsmeyer-Peppas Equation (4)		Peppas-Sahlin Equation (5)			
	10^2^·*k*_11_ (min^−1^)	AIC	10^2^*·k_h_* (min^−1/2^)	AIC	*n*	AIC	10·*k*_1_ (min^−^*^m^*)	10^2^*·k*_2_ (min^−2^*^m^*)	*m*	AIC
1	2.250	−78.600	2.588	−73.785	0.831	−34.492	1.446	0.778	0.465	−108.299
2	0.984	−71.619	1.294	−111.686	0.863	−31.354	1.896	3.274	0.744	−51.282
3	3.274	−84.467	1.422	−150.85	0.672	−37.223	1.659	2.642	0.186	−109.711
4	0.431	−46.850	7.428	−44.058	0.806	−41.852	2.337	1.678	0.713	−90.105
5	2.590	−42.543	7.328	−46.382	0.835	−43.257	2.352	2.190	0.843	−116.873
6	1.375	−89.8648	2.040	−96.243	0.770	−74.9818	5.061	6.918	0.120	−105.87

## Data Availability

The data are available on request.
